# A New Onset of Ulcerative Colitis Post-COVID-19: A Case Report

**DOI:** 10.7759/cureus.36257

**Published:** 2023-03-16

**Authors:** Chenfan Xia, Jayanthi Dissanayake, David Badov

**Affiliations:** 1 Department of Medicine, Frankston Hospital, Melbourne, AUS; 2 Pathology, Dorevitch Pathology, Melbourne, AUS; 3 Department of Gastroenterology, Frankston Hospital, Melbourne, AUS

**Keywords:** new onset diseases post covid-19, auto-imune diseases, bloody diarrhea, sars-cov-2 (severe acute respiratory syndrome coronavirus -2), covid 19, inflammatory bowel disease, ulcerative colitis (uc)

## Abstract

Severe acute respiratory syndrome coronavirus-2 (SARS-CoV-2) can cause not only respiratory symptoms but also gastrointestinal symptoms. In addition, there is increased concern about the autoimmune complications of coronavirus disease 2019 (COVID-19). This report describes a 21-year-old non-smoking Caucasian male with a history of acute pancreatitis but no other medical issues or family history who developed a new onset of ulcerative colitis after the second episode of COVID-19. He had three doses of the BNT162b2 mRNA COVID-19 vaccine. Two months after the first episode of COVID-19, he had the third dose of the vaccine. Nine months after the third dose, he had the second episode of COVID-19, during which he was mildly unwell for three days, recovered, and did not require any anti-viral medication or antibiotics. One week post the second episode of COVID-19, he developed diarrhoea and abdominal pain. It then progressed to bloody diarrhea. We diagnosed ulcerative colitis based on his clinical symptoms, biopsy changes, and the exclusion of other causes. This case raises awareness of developing ulcerative colitis concurrently with or following COVID-19. It is essential to thoroughly investigate COVID-19 patients who have diarrhea or bloody diarrhea and not consider it a common gastroenteritis or a simple gastrointestinal manifestation of COVID-19. Although we cannot confirm the association with a case study, further research is needed to confirm the causal or incidental relationship and observe any increased incidence of ulcerative colitis in the future as secondary to COVID-19.

## Introduction

Coronavirus disease 2019 (COVID-19) is an infectious disease caused by severe acute respiratory syndrome coronavirus-2 (SARS-CoV-2). Most people infected with SARS-CoV-2 will experience respiratory symptoms. It can also affect other organ systems. Up to 20% of patients reported having gastrointestinal symptoms such as diarrhoea, nausea, vomiting, and abdominal pain [1]. In addition, there is increased concern about its autoimmune complications [2]. Some patients develop autoimmune diseases such as Guillain-Barre syndrome, systemic lupus erythematosus, autoimmune haemolytic anemia, or autoimmune thyroid disease [2,3]. Ulcerative colitis is an inflammatory bowel disease (IBD) characterized by colonic epithelial cell damage [4]. The involvement of autoimmunity has been suggested in its pathogenesis [4]. This report describes the case of a 21-year-old male who developed gastrointestinal symptoms, including bloody diarrhoea, soon after the second episode of SARS-CoV-2 infection. We diagnosed ulcerative colitis based on his clinical symptoms, biopsy changes, and the exclusion of other causes.

## Case presentation

A 21-year-old Caucasian male nursing student had a history of acute pancreatitis with an unclear aetiology two years ago. He takes ibuprofen occasionally for headaches, but has no other medical issues and is not on any regular medication. He is a non-smoker and drinks alcohol occasionally. His mother has hypothyroidism with an unclear cause. There is no family history of IBD.

He had a total of three doses of the BNT162b2 mRNA COVID-19 vaccine. The first dose of the vaccine was given in August 2021. Then he had the second dose in September 2021. In January 2022, he had his first episode of COVID-19. Although not hypoxic, he felt pretty unwell for a week, with a severe cough, fever, generalized body ache, headache, and loss of taste. He received the third dose of the vaccine in March 2022.

In December 2022, he had the second episode of COVID-19. His partner tested positive first. Then he had a positive rapid antigen test two days later. He was mildly unwell for three days with a sore throat, headache, and lethargy, but then recovered. He did not require any antiviral medication or antibiotics.

One week after COVID-19, he developed diarrhoea, up to six times a day, associated with abdominal pain. After two weeks of diarrhea, he presented to the emergency department (ED). He was afebrile. Full blood count and C-reactive protein (CRP) were normal (Table 1).

**Table 1:** Comparison of lab investigations with the first and second ED presentations.

Lab investigations	First ED presentation	Second ED presentation	Normal range
Haemoglobin (g/L)	141	141	130–180
Platelet (× 10^9^/L)	233	263	150–450
White cell count (× 10^9^/L)	7.6	6.8	4.0–11.0
C-reactive protein (mg/L)	7	57	0–10

Lipase was less than 10 U/L (normal, 0-60 U/L). It was thought to be gastroenteritis, although stool microscopy and culture were negative. He was discharged home without treatment. One week after the discharge, he presented with worsening diarrhea with mucus and blood in the stool and constant abdominal pain, despite taking ibuprofen once or twice a day at home. He has no extra-intestinal symptoms. On examination, he appears lethargic. The abdomen was soft, with mild tenderness on the lower abdomen on palpation.

**Figure 1:**
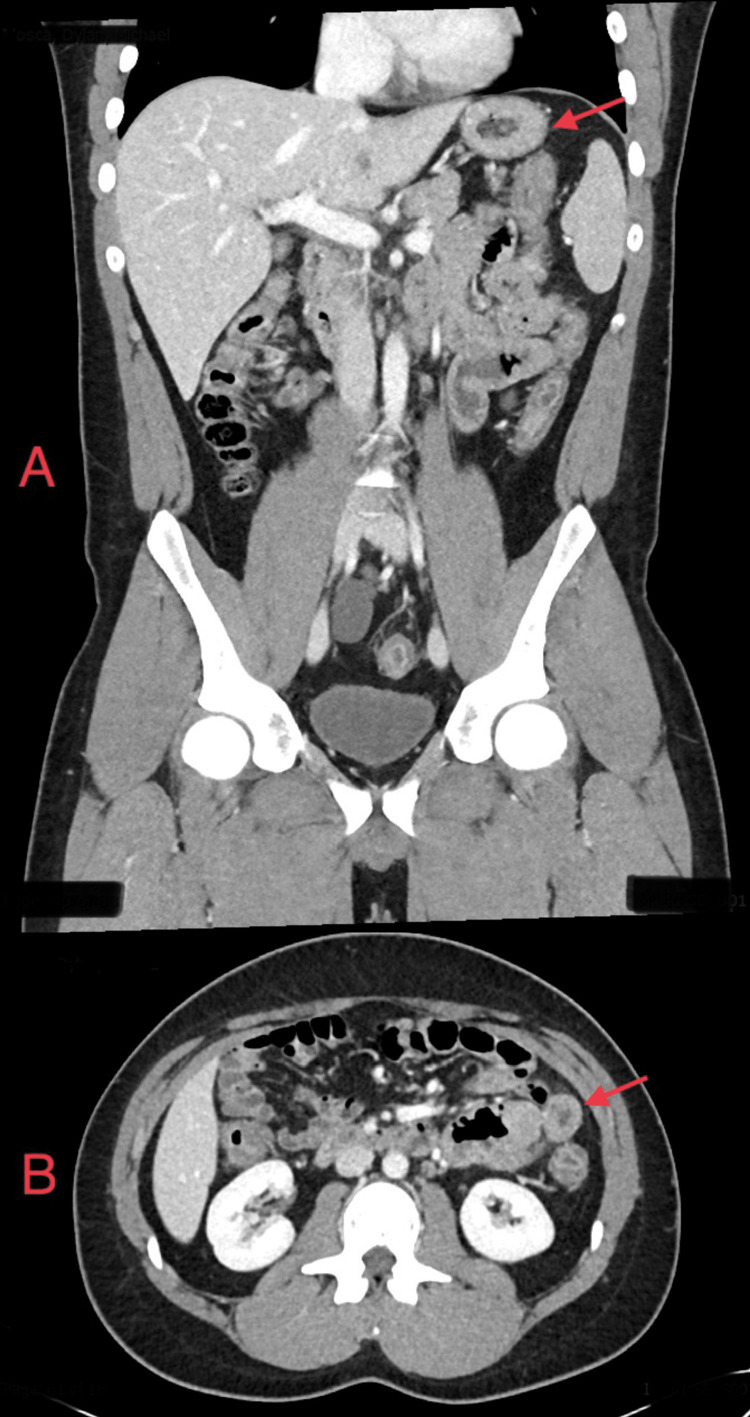
CT abdomen shows circumferential wall thickening of the colon (red arrows).

An investigation showed CRP increased to 57 mg/L (normal 0-10 mg/L) and faecal calprotectin was high at 649 µg/g (normal <50 µg/g). Computerized tomography (CT) abdomen showed circumferential wall thickening with vascular congestion involving the transverse colon, ascending colon, sigmoid, and rectum, consistent with pancolitis (Figure 1).

The full blood count was similar to one week ago (Table 1). The erythrocyte sedimentation rate (ESR) was 13 mm/hr (normal < 15 mm/hr). He had investigations to exclude other infectious causes for colitis, which were all negative. Epstein-Barr virus immunoglobulin G (IgG) antibodies were positive, but the immunoglobulin M (IgM) antibodies were negative. Cytomegalovirus (CMV) IgG and IgM antibodies were both negative. He had a repeat stools microscopy and culture. Giardia and Cryptosporidium were not detected. No Salmonella, Shigella, or Campylobacter species were isolated on culture. Faecal viral polymerase chain reaction (PCR) was negative for norovirus, rotavirus, and adenovirus. The *Clostridium difficile* toxin was not detected by PCR. The quantiferon gold test for tuberculosis was negative. The human immunodeficiency virus (HIV) test was negative.

He also had investigations to look for associated autoimmune conditions, which were all unremarkable. Autoimmune screening, including antinuclear antibodies (ANA), extractable nuclear antigens (ENA), and anti-double strand DNA (anti-DsDNA), were all negative. The human leukocyte antigen B27 (HLA-B27) allele was not detected.

**Figure 2:**
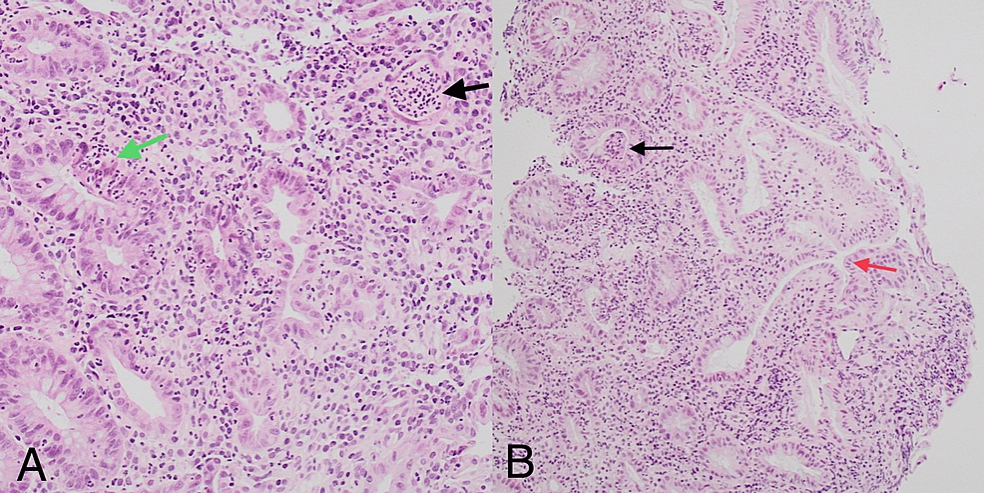
Pathology slides from biopsy show active colitis. (A) Increased lymphoplasmacytic and neutrophilic infiltration in lamina propria, cryptal abscess with neutrophil within the lumen of crypt (black arrow) and cryptitis with neutrophil within epithelial cells (green arrow), shown at 100× magnification with hematoxylin and eosin (H&E) stain. (B) Crypt architectural distortion with branching (red arrow) and cryptal abscess with neutrophil within the lumen of crypt (black arrow), shown at 40× magnification with hematoxylin and eosin (H&E) stain.

He had a flexible sigmoidoscopy. The mucosa of the examined colon appeared erythematous and oedematous with superficial ulceration and loss of vascular pattern, suggesting moderate colitis with a Mayo score of 2. Unfortunately, images were not captured. Biopsies were taken. CMV immunohistochemistry was negative. Histology showed severely active, chronic colitis with focal ulceration, consistent with inflammatory bowel disease (Figure 2).

His Mayo score for ulcerative colitis disease activity was 9 out of 12 overall, including a score of 3 for stool frequency (>4 stools/day more than normal), a score of 2 for rectal bleeding (visible blood with stools half of the time or more), a score of 2 for a mucosal appearance at endoscopy (moderate disease), and a score of 2 for the physician's rating of disease activity (moderate). He was treated with intravenous hydrocortisone 100 mg every six hours, mesalazine tablets (2 g daily for six days), and mesalazine rectal enema (1 g per 100 ml daily for three days). His symptoms improved. He was then changed to azathioprine 50 mg daily and prednisolone 40 mg daily with a weaning plan and discharged home.

We reviewed him in the outpatient clinic two months post-discharge. He felt well and had occasional urgency and some flatulence, but no further episodes of loose stools. His prednisolone was weaned and ceased. We increased azathioprine to 150 mg daily and restarted mesalazine tablets at 1 g twice a day. He will need ongoing follow-up and possibly a repeat endoscopy to monitor his progress.

## Discussion

A few theories explain the link between COVID-19 and IBD. Some studies mentioned the increased cytokines [5] and T-cell response [6] in COVID-19, suggesting a potential association with autoimmunity. However, our patient's autoimmune antibodies were all negative. Angiotensin-converting enzyme 2 (ACE 2) expression and activity were lower in a colonic biopsy in patients with active IBD than in patients with inactive disease status [7]. While SARS-CoV-2 binding to the ACE2 receptors leads to internalization and reduced surface expression of ACE2 [8]. Dysregulation of gut microbiota may also play a role. One study showed hospitalized patients with COVID-19 infection had a persistent alteration of the gut microbiome compared to uninfected controls, characterized by increased opportunistic pathogens and decreased beneficial commensals [9]. In addition, males are more likely to develop ulcerative colitis than females, possibly due to sex-specific differences in the immune system and the hormonal influence on the inflammatory response [10].

There are ten published case reports about the newly diagnosed ulcerative colitis associated with the SARS-CoV-2 infection. The patient's age ranges between 19 and 84. Three were female cases [11-13], and seven were male cases [14-19]. Our case involved a 21-year-old male patient. In the ten published cases, gastrointestinal symptoms happened concurrently with or up to three months post-COVID-19, while our patient's symptoms started one week post-COVID-19 (Table 2).

**Table 2:** Case reports of ulcerative colitis related to recent COVID-19 in chronological order.

Referecence number	Gender	Age (years)	Time of onset	Symptoms	Covid-19 treatment
11	Female	19	Same time as COVID-19	Bloody diarrhea	Hydroxychloroquine
12	Female	Young (age not mentioned in the report)	Same time as COVID-19	Bloodless watery diarrhea initially, then bloody diarrhea 4 months later	hydroxychloroquine, lopinavir/ritonavir, azithromycin
13	Female	71	Same time as COVID-19	Bloody diarrhea	Not mentioned in the report
14	Male	84	Same time as COVID-19	Septic shock, diarrhea	Vancomycin and Piperacillin/tazobactam initially, then changed to ciprofloxacin and metronidazole
Our case	Male	21	1 week post COVID-19	Bloodless diarrhea initially, then bloody diarrhea 2 weeks later	None
15	Male	64	1 week post COVID-19	Diarrhea initially, then bloody diarrhea 2 weeks later	None
16	Male	50	3 weeks post COVID-19	Bloody diarrhea	Hydroxychloroquine, azithromycin
17	Male	74	1 month post COVID-19	Bloody diarrhea	none
18	Male	18	1 month post COVID-19	Bloody diarrhea	Not mentioned in the report
15	Male	37	2 months post COVID-19	Bloody diarrhea	Steroids
19	Male	55	3 months post COVID-19	Bloody diarrhea	Steroids, azithromycin, heparin

## Conclusions

This case described a 21-year-old male patient who developed a new onset of ulcerative colitis post the second episode of COVID-19. Interestingly, he had the first episode of COVID-19 11 months before the second episode, but the gastrointestinal symptoms started abruptly one week post the second SARS-CoV-2 infection. He was diagnosed with ulcerative colitis post-flexible sigmoidoscopy and biopsy. This report raises awareness of developing ulcerative colitis concurrent with or following COVID-19. It is essential to thoroughly investigate COVID-19 patients who have diarrhea or bloody diarrhea and not consider it a common gastroenteritis or a simple gastrointestinal manifestation of COVID-19. Although we cannot confirm the association with a case study, further research is needed to confirm the causal or incidental relationship and observe any increased incidence of ulcerative colitis in the future as secondary to COVID-19.
